# New species and new records of *Manota* Williston from Colombia, Brazilian Amazonia, and Costa Rica (Diptera, Mycetophilidae)

**DOI:** 10.3897/zookeys.668.11350

**Published:** 2017-04-13

**Authors:** Olavi Kurina, Heikki Hippa, Dalton de Souza Amorim

**Affiliations:** 1 Institute of Agricultural and Environmental Sciences, Estonian University of Life Sciences, Kreutzwaldi st 5D, 51014 Tartu, Estonia; 2 Heikki Hippa, Zoological Museum, Department of Biology, FI-20014 University of Turku, Finland; 3 Departamento de Biologia, Faculdade de Filosofia, Ciências e Letras de Ribeirão Preto, Universidade de São Paulo, Av. Bandeirantes, 3900, 14040-901, Ribeirão Preto, SP, Brazil

**Keywords:** Diptera, *Manota*, Neotropical region, new species, Sciaroidea, taxonomy

## Abstract

The following five species are described as new: *Manota clava* **sp. n.** (Colombia), *Manota multilobata* **sp. n.** (Colombia), *Manota perplexa* **sp. n.** (Costa Rica), *Manota setilobata* **sp. n.** (Colombia) and *Manota subaristata* **sp. n.** (Colombia). In addition, new records for the following 11 species are presented: *Manota acuminata* Jaschhof & Hippa, 2005 (Costa Rica), *Manota arenalensis* Jaschhof & Hippa, 2005 (Costa Rica), *Manota corcovado* Jaschhof & Hippa, 2005 (Costa Rica), *Manota costaricensis* Jaschhof & Hippa, 2005 (Costa Rica), *Manota diversiseta* Jaschhof & Hippa, 2005 (Colombia, Brazilian Amazonia, Costa Rica), *Manota minutula* Hippa, Kurina & Sääksjärvi, 2017 (Brazilian Amazonia), *Manota multisetosa* Jaschhof & Hippa, 2005 (Costa Rica), *Manota parva* Jaschhof & Hippa, 2005 (Colombia, Costa Rica), *Manota pisinna* Hippa & Kurina, 2013 (Brazilian Amazonia), *Manota spinosa* Jaschhof & Hippa, 2005 (Colombia) and *Manota squamulata* Jaschhof & Hippa, 2005 (Costa Rica). Distribution patterns include (1) species known only locally in Costa Rica or Colombia, (2) distributions connecting Central America to west Andes lowlands, and (3) north-west Neotropical components, extending from Central America to Brazilian Amazonia. The possible biogeographical and taxonomical context of *Manota* species with a widespread distribution is considered.

## Introduction

The monophyletic subfamily Manotinae of Mycetophilidae is represented by four extant genera in the world fauna, but only *Manota* Williston (type species *Manota defecta* Williston) has almost cosmopolitan distribution, with the highest diversity in tropical areas (e.g. Kurina and Hippa 2015 and references therein). The other three genera are restricted either only to the Oriental region (in case of *Paramanota* Tuomikoski and *Promanota* Tuomikoski) or to the Oriental and Australasian regions (in case of *Eumanota* Edwards) (Søli 2002, Papp 2004, Hippa and Ševčík 2010, Hippa et al. 2005, 2016). The Oriental region could be an original area of distribution of the manotines (Jaschhof et al. 2011). *Manota* is considered to be the sister group of a clade including the other three genera (Hippa et al. 2005, Ševčík et al. 2013). Members of *Manota* have a unique habitus, including small size, yellowish to brownish coloration, and considerably reduced wing venation, which makes them easily recognizable in samples (for characteristic general facies, see, e.g., Hippa and Kurina 2012, Hippa et al. 2017).

The last 15 years revealed an explosion in the number of described *Manota* species. The number of species of the genus in the world increased from 28 (Bechev 2000) to 271 (Hippa et al. 2017). In the Neotropical region, this number moved from three species (Papavero 1978) to 67 species known to date, viz. 32 species from Peru (Hippa et al. 2017), 27 species from Costa Rica (Jaschhof and Hippa 2005), 21 species from Ecuador (Hippa and Kurina 2013), 8 species from French Guyana (Hippa and Kurina 2013), four species from Mexico (Hippa and Huerta 2009), two from Brazil (Enderlein 1911, Lane 1948), and one species from each St. Vincent, Lesser Antilles (Williston 1896), Nicaragua (Hippa and Kurina 2013) and Argentina (Hippa and Kurina 2013). Most of the species are known from their type localities only. The genus was mentioned as occurring in Colombia by Oliveira and Amorim (2016); the details on the species are being dealt with in this paper. A considerable number of species from the Atlantic Forest of Brazil are to be described soon, increasing the diversity of the genus in the region.

The aim of this study is to increase the knowledge of the genus *Manota* in the Neotropical region by describing new species and by giving new records based on material collected in Colombia, Brazilian Amazonia, and Costa Rica.

## Materials and methods

The Colombian material was collected by Malaise traps within the framework of a collection project leaded by Dr. M. Sharkey (National Science Foundation Grant DEB-0205982; see also Oliveira and Amorim 2016). This project resulted in a huge amount of material, including fungus gnats, of which only a few genera have been worked to date (e.g., Oliveira and Amorim 2012, 2016, Kurina and Oliveira 2015). The Costa Rican material was collected by Malaise traps or sweeping from rainforest near the Soltis Center for Research and Education, San Isidoro. The material from Brazil comes from Malaise traps at the Reserva Ducke, in Manaus, State of Amazonas, and at the State of Roraima, close to the border with Venezuela.

All the material was initially stored in ethyl alcohol. In most cases, the hypopygium was detached from the specimen and macerated in warm 20% potassium hydroxide (KOH). Several specimens, especially those collected in Colombia, were faded after being more than a decade in alcohol. After macerating in KOH and washing in distilled water, the hypopygium was stained with Chlorazol Black and thereafter mounted in “Euparal” between two pieces of coverslip, which allowed a study from both sides under a compound microscope. These preparations are now attached to a normal microscope slides by two strips of adhesive tape across their edges and are easily detached when needed, together to the remainder of the body, which was not macerated, but dehydrated and mounted in “Euparal” under a coverslip.

**Figure 1.**
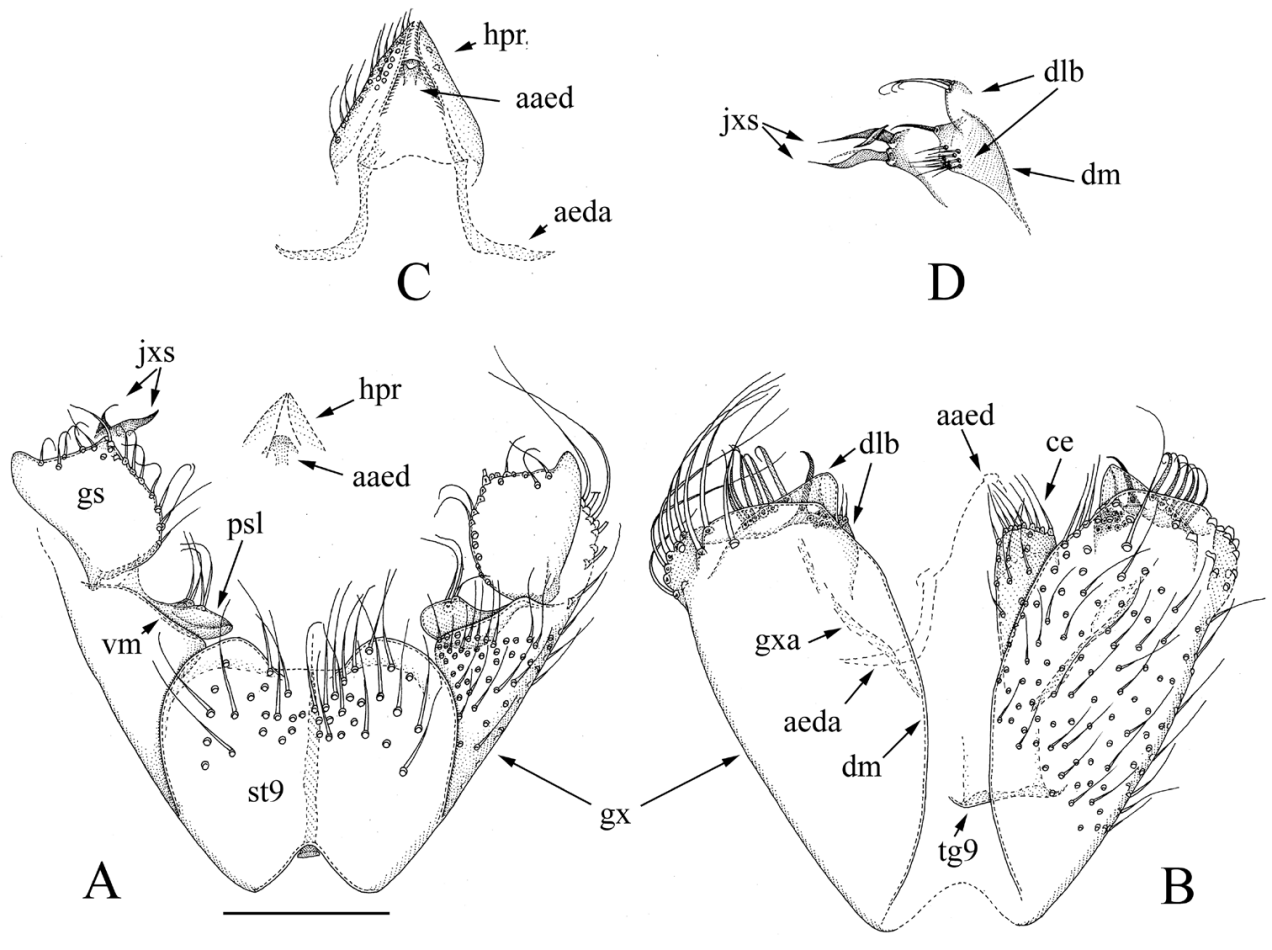
*Manota clava* sp. n. (holotype). **A** Hypopygium, ventral view **B** Hypopygium, dorsal view **C** Aedeagus and hypoproct, ventral view **D** Juxtagonostylar megasetae with associated parts, mediodorsal view. Scale bar 0.10 mm. Abbreviations: aaed = apex of aedeagus, aeda = aedeagal apodeme,ce = cercus, dlb = plates posteriorly at dorsal medial margin of gonocoxa, dm = dorsal medial margin of gonocoxa, gs = gonostylus, gx = gonocoxa, gxa = gonocoxal apodeme, hpr = hypoproct, jxs = juxtagonostylar megaseta, psl = parastylar lobe, st9 = sternite 9, tg9 = tergite 9, vm = ventral medial margin of gonocoxa.

**Figure 2.**
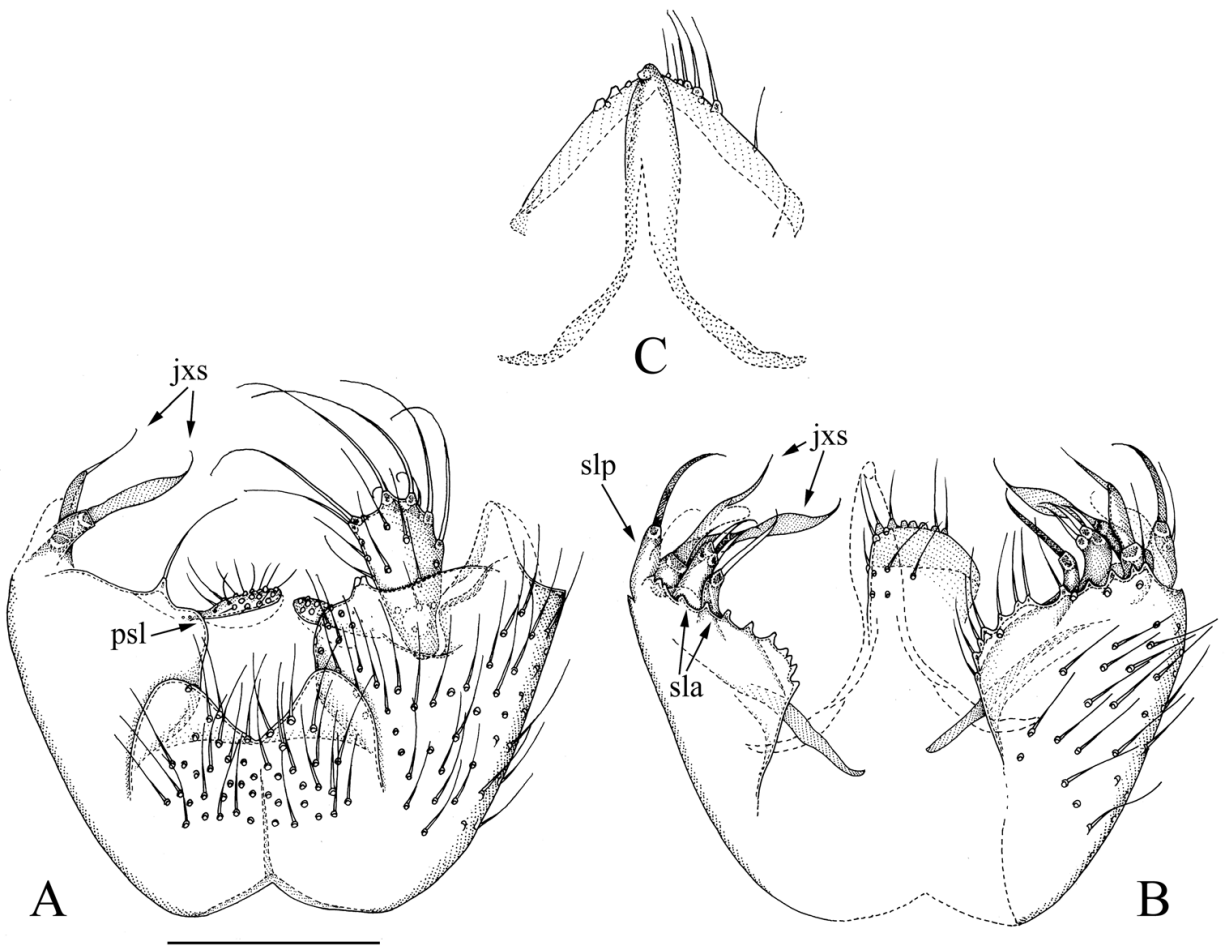
*Manota multilobata* sp. n. (holotype). **A** Hypopygium, ventral view **B** Hypopygium, dorsal view **C** Aedeagus and hypoproct, ventral view. Scale bar 0.10 mm. Abbreviations: jxs = juxtagonostylar megaseta, psl = parastylar lobe, sla = setigerous finger-like lobe anteriorly from juxtagonostylar megasetae, slp = setigerous finger-like lobe posteriorly from juxtagonostylar megasetae.

**Figure 3.**
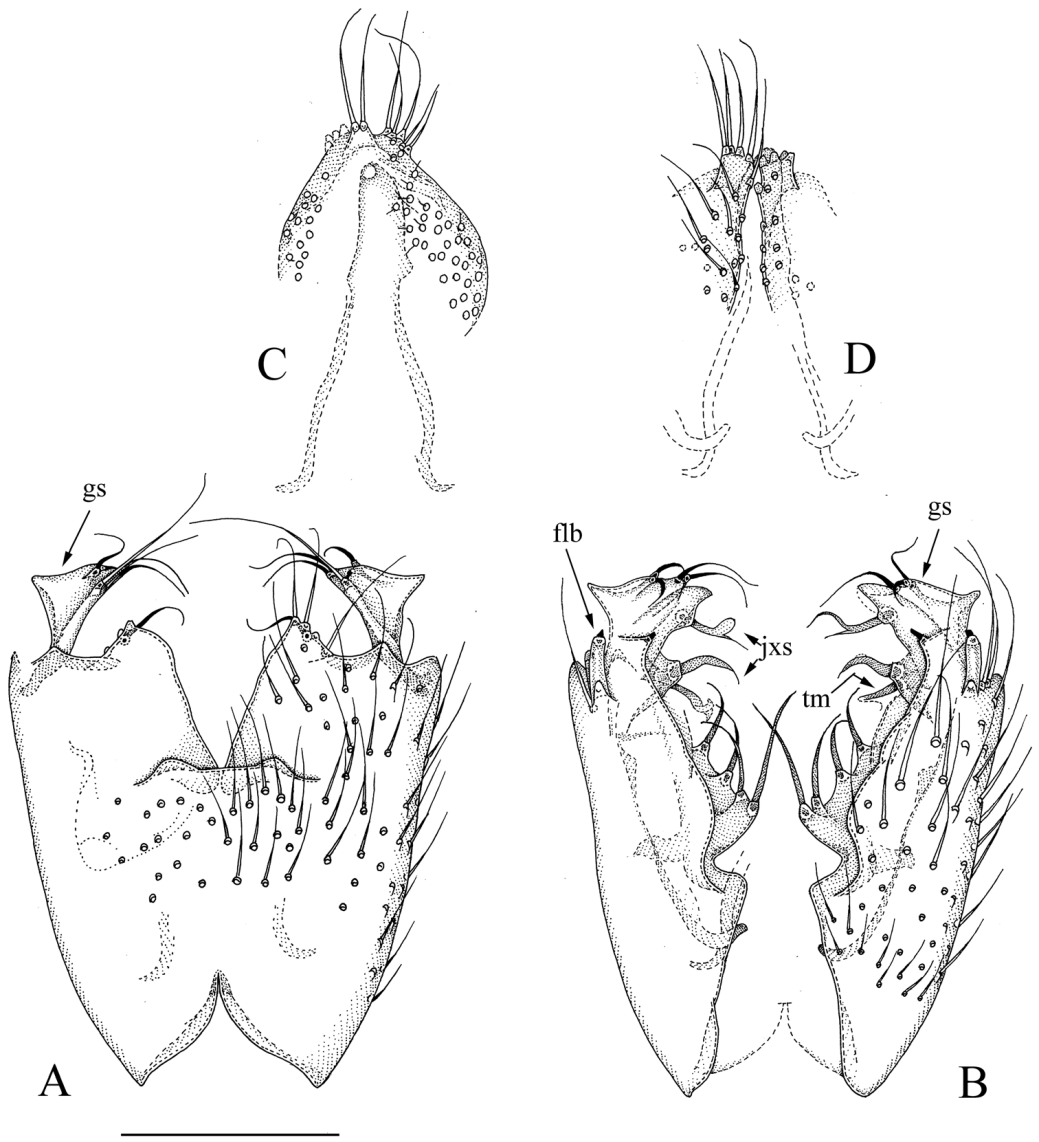
*Manota perplexa* sp. n. (holotype). **A** Hypopygium, ventral view **B** Hypopygium dorsal view **C** Aedeagus and hypoproct, ventral view **D** Cerci with associated parts, dorsal view. Scale bar 0.10 mm. Abbreviations: flb = finger-like lobe, gs = gonostylus, jxs = juxtagonostylar megasetae, tm = twisted megaseta.

**Figure 4.**
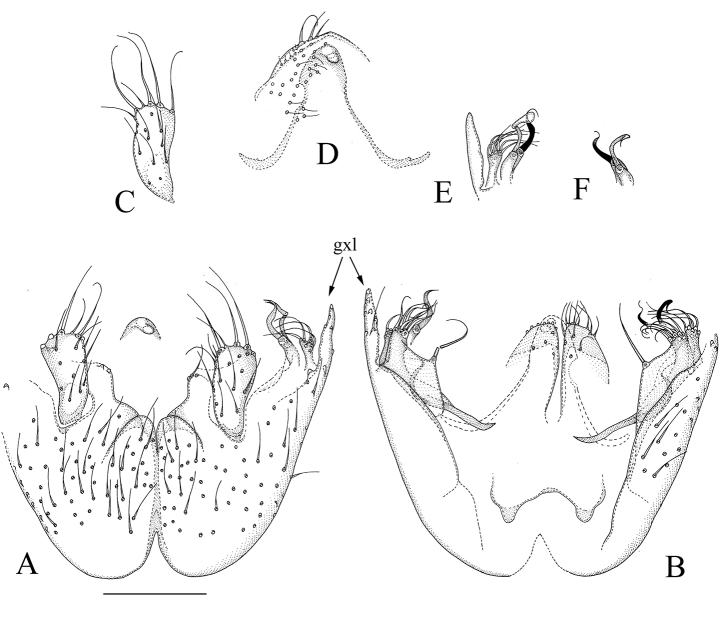
*Manota setilobata* sp. n. (**A, B, D, E** and **F** holotype, **C** paratype). **A** Hypopygium, ventral view **B** Hypopygium, dorsal view **C** Right gonostylus, ventral view **D** Aedeagus and hypoproct, ventral view **E** Left side juxtagonostylar megasetae with associated parts, dorsal view **F** Right side juxtagonostylar megasetae. Scale bar 0.10 mm. Abbreviation: gxl = posterolateral lobe of gonocoxa.

**Figure 5.**
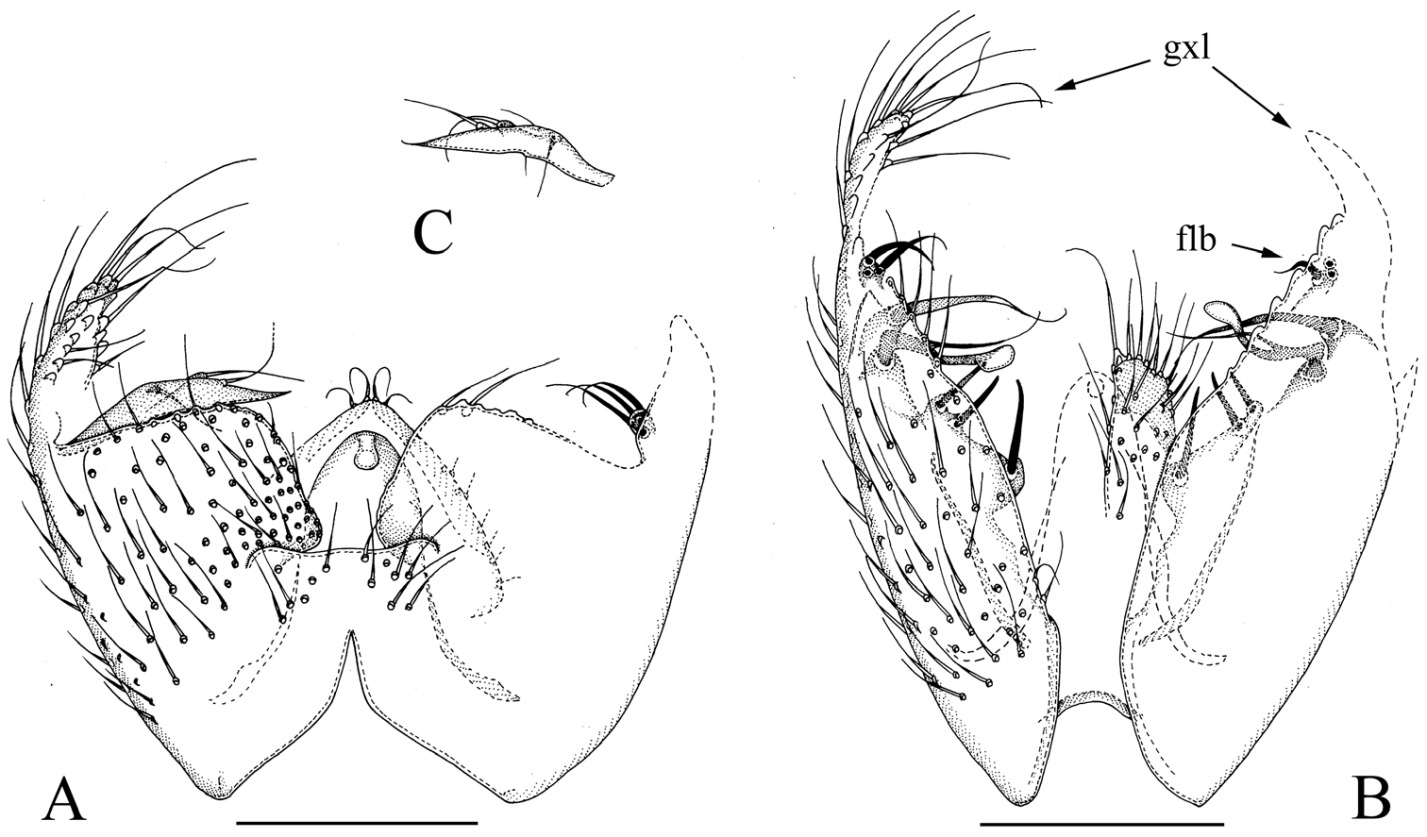
*Manota subaristata* sp. n. (**A** and **C** holotype **B** paratype). **A** Hypopygium, ventral view **B** Hypopygium, dorsal view **C** Right gonostylus, ventral view. Scale bar 0.10 mm. Abbreviations: flb = finger-like setigerous lobe, gxl = posterolateral lobe of gonocoxa.

The morphological terminology follows mainly Søli et al. (2000), while the term “parasegment” is used in accordance with Jaschhof and Hippa (2005). The terminology of the hypopygium follows Hippa & Papp (2007), but the term aedeagus is used here instead of tegmen. The terminology of hypopygium is explained in Figs 1–5. The mid tibial organ is an area of tightly placed setae basoventrally on the mid tibia (Jaschhof and Jaschhof 2010). The hind tibial organ is a similar area apicoventrally on the hind tibia (Jaschhof et al. 2011). Wing length was measured from wing base to wing tip. Description of colour was made from specimens on slides under a stereomicroscope; when available, additional specimens in ethanol were used to confirm sclerite colours. In slides, medial part of the scutum, scutellum and abdominal tergites appear somewhat darker due to their curvature on slide, while they are unicolorous in ethanol preserved specimens. Illustrations were made with the aid of a drawing tube attached to a Leitz Diaplan compound microscope. The slide mounting was done under a Leica MZ16 stereomicroscope; compound microscopes Leica DM 2500 and Leica DM 6000 B were used for final identification of species.

The material is deposited in the following collections:

**IAvH** Alexander von Humboldt Biological Resources Research Institute, Bogota, Colombia;

**IZBE** Institute of Agricultural and Environmental Sciences, Estonian University of Life Sciences (formerly Institute of Zoology and Botany), Tartu, Estonia;

**MNCR** InBio collection, Museo Nacional de Costa Rica, San José, Costa Rica;

**MZUSP** Museu de Zoologia da Universidade de São Paulo, São Paulo, Brazil.

## Taxonomy

### Manota

Taxon classificationAnimaliaDipteraMycetophilidae

Williston

Manota Williston, 1896: 260. Type-species, M. defecta Williston (mon.).
Aphanizophleps Enderlein, 1911: 201. Type-species, A. coxata Enderlein (orig. des.).

#### References.

Hippa et al. 2005 (phylogeny); Jaschhof and Hippa 2005 (identification key to Costa Rican species); Hippa and Huerta 2009 (new species from Mexico); Hippa and Kurina 2013 (new species from Ecuador, French Guyana, Nicaragua, Argentina and Peru); Oliveira and Amorim 2014 (catalogue of Neotropical Mycetophilidae); Hippa et al. 2017 (new species from Peru).

### Manota clava sp. n.

Taxon classificationAnimaliaDipteraMycetophilidae

http://zoobank.org/7B2C94AD-B520-4D59-B86F-B9099E7FB946

Figs 1A–D , 6

#### Types.

*Holotype.* Male, COLOMBIA, Risaralda, SFF Otún Quimbaya Cuchilla Camino, 04°43'N, 75°35'W, 2050 m, Malaise trap, 03–19.i.2003, G. López Leg. M. 3702 (on slide, IAvH).

#### Diagnosis.

Laterotergite setose; anterior basalare non-setose; sternite 9 posteriorly broadly concave and laterally free from gonocoxa; parastylar lobe large and apically broadened; gonocoxa without a remarkable posterolateral lobe; gonostylus subrectangular, posterolaterally drawn out; two juxtagonostylar megasetae, ventral one flame-shaped and pointed, dorsal one bilobed.

#### Description.

Male. **Colour.** Head brown, face somewhat paler. Antenna light brown, including scape and pedicel. Clypeus and mouthparts pale yellow. Thorax light brown. Legs yellowish. Wing with light brownish tinge because of microtrichia; halter brownish with blackish knob. Abdomen with tergites dark brown to blackish, sternites light brown to yellowish. All vestiture pale, yellowish or brownish, thicker setae and trichia seeming darker than finer ones. **Head.** Antennal flagellomere 4 ca. 2 times as long as wide. Palpomere 3 of maxillary palpus with apicomesial thumb-like extension, with 3 apically curved sensilla; palpomere 4 with parasegment; palpomere 5 ca. 1.1 times longer than palpomere 4. Number of strong postocular setae, 9. **Thorax.** Anepisternum with 52 setae; anterior basalare and preepisternum 2 non-setose; laterotergite with 13 setae; metepisternum with 9 setae. **Legs.** Mid and hind tibial organs absent. **Wing.** R_1_ meeting C within basal half of costal margin; sclerotized part of M_2_ extending to level of tip of R_1_; wing length, 2.4 mm. **Hypopygium** (Fig. 1A–D). Sternite 9 ca. 2/3 as long as gonocoxa with delimited lateral margins, broadly concave posteriorly, anteriorly incised; posterior half covered with setae which are slightly stronger than adjacent ventral setae of gonocoxa, anterior half non-setose. Ventral medial margin of gonocoxa simple. Parastylar lobe large, apically broadened, club-like, with 3-5 fine posteriorly directed setae medially. No paraapodemal lobe observable. Posterolateral part of gonocoxa not drawn into a remarkable lobe but bearing many long curved setae. Dorsal medial margin of gonocoxa simple, medially bulging, posteromedially almost right-angled. Ventrally from posteromedial corner, there are two plate-like lobes on different levels: more dorsal lobe bears 6 strong apically curved setae laterally, more ventral lobe bears aggregation of fine setae at anteromedial corner and one strong seta at posteromedial corner. Two juxtagonostylar megasetae present, the more anterior and ventral one flame-shaped and pointed, with obscurely discernible seta-like branch marked by broken line in Fig. 1D, the more posterior and dorsal one bilobed, both arising from a large common basal body which is as long as megasetae. Gonostylus sub-rectangular, posterolateral corner drawn out into a rounded lobe, posterior and medial margin with short setae. Aedeagus broadly subtriangular, with lateral shoulders, the apex curved ventrally. Aedeagal apodemes directed laterad. Hypoproct extending posteriorly to apex of gonostyli, each side with ca. 25 normal setae on ventral surface. Cerci medially separated.

Female. Unknown.

#### Discussion.

The setose laterotergite, non-setose anterior basalare, sternite 9 laterally free from gonocoxa, and gonocoxa without a remarkable posterolateral lobe group together *Manota clava* sp. n. with *M. caribica* Jaschhof & Hippa, 2005 (Costa Rica) and *M. micula* Hippa & Kurina, 2013 (Ecuador, Peru). All three species have also the sternite 9 posteriorly broadly concave and a similar aggregation of setae on plate-like lobe ventrally from dorsal medial margin of gonocoxa. Parastylar lobe is distinct between all three species: large, apically broadened with 3–5 posterior setae in *M. clava*, large, subtriangular with three posterior setae in *M. micula* and small, stout with 2–3 setae posteroapically in *M. caribica*. The gonostylus of *M. clava* is subrectangular and posterolaterally drawn out while it is oval or almost circular in case of the two other species. *Manota clava* and *M. micula* have the juxtagonostylar megasetae complex with transverse and leaf-like expansions, while they are simple and pointed in *M. caribica*.

#### Etymology.

The specific epithet is Latin, *clava* [club or mace], referring to the prominent club-shaped parastylar lobe, and is a noun used as in apposition.

### Manota multilobata sp. n.

Taxon classificationAnimaliaDipteraMycetophilidae

http://zoobank.org/DF26817D-97B1-408E-B9F9-875D7F5688C9

Figs 2A–C , 6

#### Types.

*Holotype.* Male, COLOMBIA, Valle de Cauca, PNN Farallones de Cali Cgto., La Meseta, 03°34'N, 76°40'W, 2,200 m, Malaise trap, 27. viii–10.ix.2003, S. Sania & M. Losso col., M 4570 (on slide, IAvH).

#### Diagnosis.

Laterotergite non-setose; anterior basalare non-setose; sternite 9 posteriorly broadly and deeply concave, anterior half fused to gonocoxa; parastylar lobe transversally oblong, with *ca* 20 setae; gonocoxa drawn into a short and broad posterolateral lobe; gonostylus widening apically, somewhat sunken into gonocoxa; two juxtagonostylar megasetae, ventral one flame-shaped, dorsal one twisted; two and one apically setose finger-like lobes anteriorly and posteriorly from juxtagonostylar megasetae, respectively.

#### Description.

Male. **Colour**. Head brown, face somewhat paler. Antenna light brown, including scape and pedicel. Clypeus and mouthparts yellowish. Thorax light brown. Legs yellowish, basal third of femur 3 infuscated. Wing with brownish tinge because of microtrichia; halter yellow with blackish knob. Abdomen with tergites brownish, sternites somewhat lighter. All vestiture pale, yellowish or brownish, thicker setae and trichia seeming darker than finer ones. **Head**. Antennal flagellomere 4 ca. 2.1 times as long as wide. Palpomere 3 of maxillary palpus with apicomesial thumb-like extension, its curved sensilla not discernible; palpomere 4 with parasegment; palpomere 5 not measurable on holotype. Number of strong postocular setae 9. **Thorax**. Anepisternum with 26 setae; anterior basalare, preepisternum 2 and laterotergite non-setose; metepisternum with 8 setae. **Legs**. Mid and hind tibial organs absent. **Wing**. R_1_ meeting C within basal half of costal margin; sclerotized part of M_2_ extending to level of tip of R_1_; wing length, 2.5 mm. **Hypopygium** (Fig. 2A–C). Sternite 9 broad, extending to the middle of gonocoxa, anterior half fused to gonocoxa, posterior half free, posterior margin broadly and deeply concave, anterior margin shallowly and angularly incised, laterally with bare narrow area, medially setose with setae similar to the adjacent ventral setae of gonocoxa. Ventral medial margin of gonocoxa simple. Posterior margin with two long setae having prominent sockets. Parastylar lobe transversally oblong, well exposed in ventral view, bearing ca 20 setae. No paraapodemal lobe observable. Posterolateral part of gonocoxa not drawn into a remarkable lobe. Dorsal medial margin of gonocoxa simple, bulging medially, contiguous with the dorsal posterior margin. Two juxtagonostylar megasetae arising from separate basal bodies, dorsal megaseta somewhat twisted, with a basal body ca. one fourth of seta’s length, ventral megaseta flame-shaped, with a basal body slightly less than seta’s length. Two finger-like lobes anteriorly from juxtagonostylar megasetae: more anterior lobe subequal to basal body of dorsal juxtagonostylar megaseta with one seta apically, more posterior lobe subequal to basal body of ventral juxtagonostylar seta with three setae apically. Posteriorly from the juxtagonostylar megasetae, a lobe, subequal to basal body of ventral juxtagonostylar seta, bearing one strong apical seta and one weak subapical seta. Gonostylus somewhat sunken into gonocoxa, apically widening, with 5 strong and long setae at posterior and posterolateral margins, other setosity similar to that on gonocoxa ventrally. Aedeagus elongate, narrowly subtriangular, the lateral sides slightly concave, apex curved ventrally. Hypoproct extending posteriorly to level of apex of gonostyli, each side with 6 setae on apical third ventrally. Cerci broad, medially separated.

Female. Unknown.

**Discussion.** *Manota multilobata* sp. n. groups together with *M. setilobata* sp. n. by having the non-setose anterior basalare, non-setose laterotergite, indistinct or short posterolateral lobes of the gonocoxa, and the megasetae and aggregations of setae at the dorsal medial margin of the gonocoxa all placed far posteriorly. Both species have the obovate gonostylus, which is somewhat sunken into the gonocoxa, and have 4–5 strong apical and subapical setae deviating from other setae, similar arrangement of small setose lobes around juxtagonostylar setae, and sternite 9 basally fused with the gonocoxa. The species differ as follows: 1) in *M. multilobata* there are two finger-like lobes close together anteriorly from the juxtagonostylar megaseta, the more anterior one with one, the more posterior one with three strong setae (in *M. setilobata* there is a plate-like lobe anteriorly bearing one seta widely separated from a posterior group of several setae), 2) in *M. multilobata* posteriorly from the juxtagonostylar megasetae there is a finger-like lobe with one strong and one weak seta (in *M. setilobata* a flat lobe with numerous fine setae), 3) in *M. multilobata* the gonocoxa is drawn into a short and broad posterolateral lobe (in *M. setilobata* it is drawn into a short and narrow lobe), and 4) in *M. multilobata* sternite 9 has the posterior margin broadly v-shaped incised (in *M. setilobata* there is narrow and deep medial cleft).

#### Etymology.

The specific epithet is Latin, *multilobata* [many-lobed], referring to the setigerous lobes dorsally on the gonostylus (adjective).

### Manota perplexa sp. n.

Taxon classificationAnimaliaDipteraMycetophilidae

http://zoobank.org/7FB344E8-9876-4EDD-8253-AD0167D702C8

Figs 3A–D , 6

#### Types.

*Holotype.* Male, COSTA RICA, San Isidro de las Peñas Blancas, Texas A&M Soltis Center, Malaise trap, 400 m, 10°23'00"N, 84°36'58"W, 20.iv–26. v.2010, Wendy Porras col. (on slide, MNCR).

#### Diagnosis.

Laterotergite non-setose; anterior basalare non-setose; sternite 9 laterally entirely fused to gonocoxa, posterior margin free with protruding posterolateral corners; parastylar lobe indistinct; gonocoxa with a large plate-like lobe bearing four simple megasetae medioventrally from dorsal medial margin and anteriorly from the juxtagonostylar setae; gnostylus subtriangular, with prominent lateral angle; two juxtagonostylar megasetae, anterior one simple and pointed, posterior one bifurcated.

#### Description.

Male. **Colour.** Head brown, face somewhat paler. Antenna light brown, scape, pedicel and two basal flagellomeres slightly paler. Clypeus and mouthparts yellowish. Thorax light brown. Legs yellowish. Wing with light brownish tinge because of microtrichia; haltere yellow with brown knob. Abdomen with tergites dark brown to blackish, sternites yellowish. All vestiture pale, yellowish or brownish, thicker setae and trichia seeming darker than the finer ones. **Head.** Antennal flagellomere 4 ca. 1.8 times as long as wide. Palpomere 3 of maxillary palpus with apicomesial thumb-like extension, with three apically curved sensilla; palpomere 4 with parasegment; palpomere 5 ca. as long as palpomere 4. Nine strong postocular setae. **Thorax.** Anepisternum with 46 setae; anterior basalare, preepisternum 2 and laterotergite non-setose; metepisternum with 15 setae on anterior part. **Legs.** Mid and hind tibial organs absent. **Wing.** R_1_ meeting C within basal half of costal margin; sclerotized part of M_2_ extending to level of tip of R_1_; wing length 1.6 mm. **Hypopygium** (Fig. 3A–D). Sternite 9 laterally entirely fused to gonocoxa, posterior margin free with protruding posterolateral corners; setae similar to adjacent ventral setae of gonocoxa. Ventral medial margin of gonocoxa simple, posteromedial corner drawn into a lobe, posterolateral part of gonocoxa not drawn into a lobe. Parastylar lobe indistinct, apparently represented by one seta by ventral medial margin of gonocoxa. No paraapodemal lobe observable. Dorsal medial margin of gonocoxa with a transverse shallow incision medially. A large plate-like lobe bearing four simple megasetae medioventrally from dorsal medial margin and anteriorly from the juxtagonostylar setae, anteriormost with its own basal body ca. 1/3 longer than others. Two juxtagonostylar megasetae present, more anterior one pointed, slightly curved simple megaseta, arising from a basal body which is shorter than the megaseta, more posterior one bifurcate, one of the branches whip-like, the other flat and dilated, arising subapically from a very prominent basal body which is longer than the megaseta itself and ca. as long as gonostylus. Ventrally from the more anterior juxtagonostylar megaseta there is an apically twisted megaseta. Dorsally at the posterior margin of gonocoxa a long finger-like lobe apically bearing a seta (in holotype the seta is broken on both sides). Gonostylus subtriangular, with prominent lateral angle, with one very strong and 2–3 weaker setae at posteromedial corner, and with one curved strong seta at posterior margin. Aedeagus narrowly subtriangular, the apex curved ventrally, otherwise the details not visible in the mount. Hypoproct posteriorly extending to the base of gonostyli, with ca. 35 ventral setae on each side. Cerci medially separated, with their apical parts narrowed.

Female. Unknown.

#### Discussion.

In the key to Costa Rican species by Jaschhof and Hippa (2005), *M. perplexa* sp. n. would run into couplet 13, because of non-setose laterotergite and absence of posterolateral lobes of gonocoxa. Due to the structure of male genitalia, *M. perplexa* is clearly different from the two included species, viz. *M multisetosa* Jaschhof & Hippa and *M. tapantiensis* Jaschhof & Hippa. *Manota perplexa* is distinguished e.g. by the lack of well-developed parastylar lobe and the presence of complicated pattern of lobes and strong setae dorsally at the medial margin of gonocoxa as well as by laterally with the gonocoxa fused sternite 9. The complex dorsomedial armature of gonocoxa is unique and easily distinguishes *M. perplexa* from any other described *Manota* species.

#### Etymology.

The specific epithet is Latin, *perplexa* [confused, complicated or ambiguous], referring to the very complex gonostylus and its juxtapositional structures (adjective).

### Manota setilobata sp. n.

Taxon classificationAnimaliaDipteraMycetophilidae

http://zoobank.org/23635692-B4C3-431D-859D-A382FBD9B89D

Figs 4A–F , 6

#### Types.

*Holotype.* Male, COLOMBIA, Risaralda, SFF Otún Quimbaya Cuchilla Camino, 04°43'N, 75°35'W, 2050 m, Malaise trap, 08–24.v.2003, G. López Leg. M. 3673 (on slide, IAvH). *Paratype.* Male, same as holotype except 04–17.ii.2003, M. 3694 (on slide, IAvH).

#### Diagnosis.

Laterotergite non-setose; anterior basalare non-setose; sternite 9 posteriorly and anteriorly deeply incised, posterior third laterally free; parastylar lobe indistinct; posterolateral part of gonocoxa drawn into a narrow lobe; dorsomedial margin of gonocoxa with a large plate-like lobe bearing one strong seta at posteromedial corner; gonostylus elongated subquadrangular, slightly sunken into gonocoxa; two juxtagonostylar megasetae, both twisted, the more dorsal one apically flattened and dilated; posteriorly from the juxtagonostylar megasetae a narrow flat apically setose lobe.

#### Description.

Male. **Colour.** Head brown, face somewhat paler. Antenna light brown, including scape and pedicel. Clypeus and mouthparts yellowish. Thorax light brown. Legs yellowish. Wing with light brownish tinge because of microtrichia; haltere yellow with blackish knob. Abdomen with tergites dark brown to blackish, sternites light brown to yellowish. All vestiture pale, yellowish or brownish, thicker setae and trichia seeming darker than finer ones. **Head.** Antennal flagellomere 4 ca. 2.3 times as long as wide. Palpomere 3 of maxillary palpus with apicomesial thumb-like extension, with three apically curved sensilla; palpomere 4 with parasegment; palpomere 5 missing in both known specimens. Number of strong postocular setae, 10. **Thorax.** Anepisternum with 29–33 setae; anterior basalare, preepisternum 2 and laterotergite non-setose; metepisternum with 3–5 setae. **Legs.** Mid and hind tibial organs absent. **Wing.** R_1_ meeting C within basal half of costal margin; sclerotized part of M_2_ extending to level of tip of R_1_; wing length, 2.4 mm. **Hypopygium** (Fig. 4A–F). Sternite 9 ca. 2/3 as long as gonocoxa, anterior 2/3 laterally fused to gonocoxa, posterior 1/3 free, posterior and anterior margins with deep incisions which separate the sclerite almost into two halves, covered with setae similar to adjacent ventral setae of gonocoxa. Ventral medial margin of gonocoxa simple. Parastylar lobe not identifiable with certainty, possible fused with gonocoxa and in Fig. 4A comprising the part visible between the posterior margin of sternite 9 and the gonostylus. No paraapodemal lobe observable. Posterolateral part of gonocoxa drawn into a narrow lobe. Dorsomedial margin of gonocoxa simple. In a more ventral level, a large plate-like lobe bearing one strong seta at posteromedial corner. Two juxtagonostylar megasetae present, both twisted, the more dorsal one apically flattened and dilated, both arising from basally fused basal bodies which are as long as the megasetae. Dorsally from juxtagonostylar megasetae, a flat apically setose lobe, connected with a thin, one seta bearing plate-like lobe anteriorly from it. Posteriorly from the juxtagonostylar megasetae a narrow flat apically setose lobe. Gonostylus elongated subquadrangular, slightly sunken into gonocoxa, with 4–5 strong and long setae at posterior margins, other setosity similar to that on gonocoxa ventrally, dorsal side non-setose. Aedeagus subtriangular, lateral sides slightly concave, apex curved ventrally. Hypoproct extending posteriorly over apex of gonostyli, each side with 4–5 strong setae apically and ca. 30 fine setae on ventral surface. Cerci medially separated.

Female. Unknown.

#### Discussion.

*Manota setilobata* sp. n. resembles *M. multilobata* sp. n. For a more detailed discussion on distinguishing characters, see above.

#### Etymology.

The specific epithet is Latin, *setilobata* [with seta-bearing lobes], referring to the apically setose lobes dorsally on the gonocoxa (adjective).

### Manota subaristata sp. n.

Taxon classificationAnimaliaDipteraMycetophilidae

http://zoobank.org/E5250B84-994F-4B05-B780-ACE895044332

Figs 5A–C , 6

#### Types.

*Holotype.* Male, COLOMBIA, Valle de Cauca, PNN Farallones de Cali Cgto., La Meseta, 03°34'N, 76°40'W, 2200 m, Malaise trap, 27.viii–10.ix.2003, S. Sania & M. Losso col., M 4570 (on slide, IAvH). *Paratypes.* 2 males, same as holotype (on slides, MZUSP); 1 male, same as holotype except 24.xii.2003–27.i.2004, M 4564 (on slide, IAvH); 1 male, same as holotype except 27.i–10.ii.2004, M 4563 (on slide, IAvH); 1 male, same as holotype except 10–25.ii.2004, M 4555 (on slide, IZBE); 1 male, COLOMBIA, Huilla, PNN Cueva de los Guácharos, Alto el Mirador, 01°38'N, 76°06'W, 1980 m, Malaise trap, 6-21.iv.2002, J. Fonseca col., M 3127 (on slide, IAvH); 1 male, COLOMBIA, Cauca, PNN Gorgona, El Saman, 02°58'N, 78°11'W, 5 m, Malaise trap, 28.ix–22.x.2001, H. Torres col., M 2457 (on slide, IZBE); 1 male, same as previous except 06–23.iii.2002, R. Duque col., M 3088 (on slide, IAvH); 1 male, COLOMBIA, Risaralda, SFF Otún Quimbaya, El Molinillo, 04°43'N, 75°34'W, 2200 m, Malaise trap, 17.ii–4.iii.2003, G. López col., M 3696 (on slide, IAvH).

#### Diagnosis.

Laterotergite non-setose; anterior basalare non-setose; sternite 9 laterally fused to gonocoxa except for posterior fifth; parastylar lobe indistinct; posterolateral part of gonocoxa drawn into a lobe; dorsomedial margin of gonocoxa with a plate-like lobe bearing one anterior and two posterior simple megasetae; gonostylus in dorsal and ventral view narrow, crescent-shaped; two juxtagonostylar megasetae, more dorsal one subbasally geniculate and apically bifurcate, more ventral one simple, slightly flattened whip-like; posteriorly from juxtagonostylar megasetae a short finger-like lobe with 3–4 strong setae.

#### Description.

Male. **Colour**. Head brown, face somewhat paler. Antenna light brown, including scape and pedicel. Clypeus and mouthparts yellowish. Thorax brown. Legs yellowish. Wing with brownish tinge because of microtrichia; halter yellow with blackish knob. Abdomen with tergites brown to dark brown, sternites somewhat lighter. All vestiture pale, yellowish or brownish, thicker setae and trichia seeming darker than finer ones. **Head**. Antennal flagellomere 4 ca. 1.6–1.7 times as long as wide. Palpomere 3 of maxillary palpus with apicomesial thumb-like extension, with 3 apically curved sensilla; palpomere 4 with parasegment; palpomere 5 ca. 1.4–1.5 times longer than palpomere 4. Number of strong postocular setae 9–11. **Thorax**. Anepisternum with 42–47 setae; anterior basalare, preepisternum 2 and laterotergite non-setose; metepisternum with 8–14 setae. **Legs**. Mid and hind tibial organs absent. **Wing**. R_1_ meeting C within basal half of costal margin; sclerotized part of M_2_ extending to level of tip of R_1_; wing length, 1.9–2.3 mm. **Hypopygium** (Fig. 5A–C). Sternite 9 laterally fused to gonocoxa except for posterior 1/5, extending to middle of gonocoxa, posterior margin slightly concave, anterior margin deeply incised. Posterior 1/3 of sternite 9 setose, otherwise non-setose, setae similar to adjacent ventral setae of gonocoxa. Medioventral margin of gonocoxa roundly angled. Parastylar not identifiable, apparently membranous and covered by gonocoxa, possibly with one seta visible at the gonocoxal margin in Fig. 5A. No paraapodemal lobe observable. Posterolateral part of gonocoxa drawn into a lobe ca. 1/3 length of gonocoxa. Mediodorsal margin of gonocoxa simple. A plate-like lobe with one anterior and two posterior simple megasetae medioventrally from dorsal medial margin and anteriorly from juxtagonostylar setae. Two juxtagonostylar megasetae present: more dorsal megaseta subbasally geniculate, apical part bifurcate, one of the branches whip-like, the other apically flattened and dilated; the more ventral megaseta simple, slightly flattened whip-like. Posteriorly from juxtagonostylar megasetae a short finger-like lobe with 3–4 strong setae. Gonostylus in dorsal and ventral view narrow, crescent-shaped (in some slides apically pointed), with 3–4 setae dorsally near lateral margin and 1 ventral seta near the medial margin. Aedeagus elongate subtriangular, without lateral shoulders, apex curved ventrally. Hypoproct extending posteriorly to level of base of gonostyli or slightly over, with ca. 25 ventral setae on each side. Cerci medially separate, apically slightly widened.

Female. Unknown.

#### Discussion.

*Manota subaristata* sp. n. is similar to *M. aristata* Hippa & Kurina, 2013 in having the dorsal juxtagonostylar megaseta with a long whip-like branch. *Manota subaristata*, however, has the megaseta subbasally geniculate, arising from a separate basal body, while it is basally straight and arising from apical half of the common basal body with the ventral juxtagonostylar seta in *M. aristata*. *Manota subaristata* has 3–4 strong setae on a finger-like setose lobe posteriorly from the juxtagonostylar megaseta, which are absent in *M. aristata*. By the latter character, the species resembles *M. acutisty*lus Jaschhof & Hippa, 2005, but the megasetae at the dorsal medial margin of gonocoxa in *M. subaristata* are longer and there are two of them in the posterior group, not three as in *M. acutistylus* (see also the discussion for *M. aristata* in Hippa and Kurina 2013: 109). In *M. subaristata*, the juxtagonostylar megasetae are subequal in length, while *M. acutistylus* has the dorsal juxtagonostylar megaseta remarkably shorter, ca. half of the length of ventral one. All these three species have the gonostylus apically tapering in dorsal and ventral view, and sternite 9 largely fused to the gonocoxa, and may compose together a small clade within the Neotropical diversity of the genus.

#### Etymology.

The specific epithet is Latin, formed from the specific epithet of *M. aristata* by the prefix *sub*- [somewhat], as a reference to the similarity of the two species (adjective).

**Figure 6.**
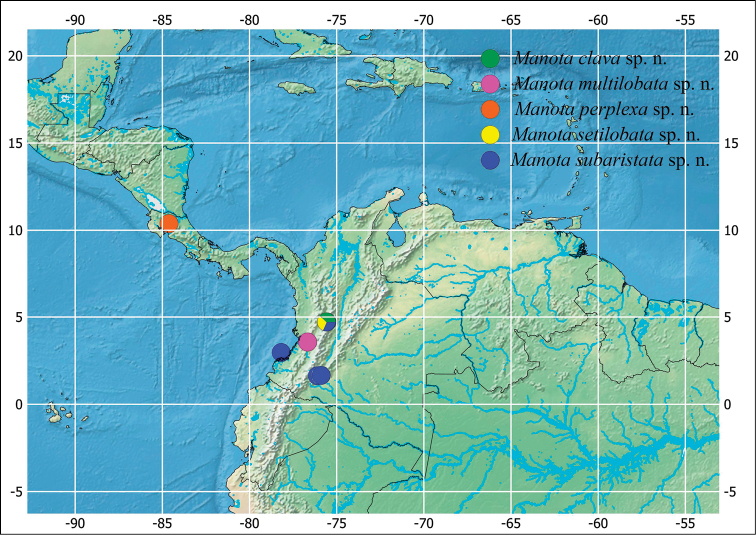
Distribution of the new species of *Manota*.

### New records

#### Manota acuminata

Taxon classificationAnimaliaDipteraMycetophilidae

Jaschhof & Hippa, 2005

##### Studied material.

COSTA RICA. 3 males, San Isidro de las Peñas Blancas, Texas A&M Soltis Center, Malaise trap, 400 m, 10°23'00"N, 84°36'58"W, 20.iv–26.v.2010, Wendy Porras col. (on slides, 1 male MZUSP, 2 males MNCR).

##### Remarks.

The species was earlier known from Costa Rica (Jaschhof and Hippa 2005), Ecuador (Hippa and Kurina 2013) and Peru (Hippa et al. 2017), hence widespread at the north-western corner of South America and Central America.

#### Manota arenalensis

Taxon classificationAnimaliaDipteraMycetophilidae

Jaschhof & Hippa, 2005

##### Studied material.

COSTA RICA. 1 male, San Isidro de las Peñas Blancas, Texas A&M Soltis Center, Sweeping, 420 m, 10°23'00"N, 84°36'58"W, 13–18.viii.2010, D. Ament col. (on slide, MZUSP); 2 males, San Isidro de las Peñas Blancas, Texas A&M Soltis Center, Malaise trap, 400 m, 10°23'00"N; 84°36'58"W, 20.iv–26.v.2010, Wendy Porras col. (on slides, MNCR).

##### Remarks.

*Manota arenalensis* was earlier known only from Costa Rica (Jaschhof and Hippa 2005).

#### Manota corcovado

Taxon classificationAnimaliaDipteraMycetophilidae

Jaschhof & Hippa, 2005

##### Studied material.

COSTA RICA. 1 male, San Isidro de las Peñas Blancas, Texas A&M Soltis Center, Sweeping, 420 m, 10°23'00"N, 84°36'58"W, 13–18.viii.2010, D. Ament col. (on slide, MNCR).

##### Remarks.

The terminalia of the specimen studied here slightly differs from those figured by Jaschhof and Hippa (2005: fig. 16): three internal megasetae on the gonocoxa subapically (= position IV by Jaschhof and Hippa 2005) are more smoothly outlined, the gonostylus is slightly wider and the apical re-curved seta on gonostylus is pointed instead of being blunt. These differences are here considered to be within intraspecific variation or are differently exposed due to different position at the slide-mounting. The large posterolateral lobes of the gonocoxa, the number and arrangement of megasetae at ventromedial margin of the gonocoxa, the shape of juxtagonostylar megasetae and sternite 9 are identical to the specimens of the original description of *M. corcovado*. The species was earlier known only from Costa Rica (Jaschhof and Hippa 2005).

#### Manota costaricensis

Taxon classificationAnimaliaDipteraMycetophilidae

Jaschhof & Hippa, 2005

##### Studied material.

COSTA RICA. 1 male, San Isidro de las Peñas Blancas, Texas A&M Soltis Center, Malaise trap, 420 m, 10°23'00"N, 84°36'58"W, 15.vi–10.vii.2010, Wendy Porras col. (on slide, MNCR).

##### Remarks.

The species is known only from Costa Rica (Jaschhof and Hippa 2005).

#### Manota diversiseta

Taxon classificationAnimaliaDipteraMycetophilidae

Jaschhof & Hippa, 2005

##### Studied material.

COLOMBIA. 1 male, Amazonas, PNN Amacayacu, Matamata, 03°41'N, 70°15'W, 150 m, Sweeping, 23.x.2000, A. Parente col., M 3552 (on slide, IAvH); 1 male, Vaupés, Estación Biológica Mosiro-Itajura (Caparú), Igapo, 01°04'S 69°31'W, 60 m, Malaise trap, 25.ii–04.iii.2003, J. Pinzón Leg. M 3627 (on slide, MZUSP). BRAZIL. 1 male, State of Amazonas, Manaus, Reserva Ducke, Igarapé Ipiranga, 2°53'S, 59°58'W, 31.xii.2002, Malaise trap, J. Vidal col. (on slide, MZUSP). COSTA RICA. 3 males, San Isidro de las Peñas Blancas, Texas A&M Soltis Center, Malaise trap, 400 m, 10°23'00" N, 84°36'58" W, 20.iv–26.v.2010, Wendy Porras col. (in alcohol, MNCR); 6 males, same data as previous except 15.vi–10.vii.2010 (4 in alcohol, MZUSP; 2 on slides, MNCR and MZUSP); 1 male, same data as previous except sweeping, 13–18.viii.2010, D. Ament col. (on slide, IZBE).

##### Remarks.

Having been described from Costa Rica (Jaschhof and Hippa 2005), the species has subsequently been recorded from Ecuador, French Guyana (Hippa and Kurina 2013) and Peru (Hippa et al. 2017).

#### Manota minutula

Taxon classificationAnimaliaDipteraMycetophilidae

Hippa, Kurina & Sääksjärvi, 2017

##### Studied material.

BRAZIL. 1 male, State of Amazonas, Manaus, Reserva Ducke, Igarapé Barro Branco, 2°59'30"S, 59°57'25"W, 12–22.vii.2004, Malaise trap, A. Henriques col. (on slide, MZUSP).

##### Remarks.

The species was earlier known only from the Iquitos area in Peru (Hippa et al. 2017). We have not been able to see much material from Manaus and this is the only species identified from the state of Amazonas, a species shared with other areas of the Amazon Basin.

#### Manota multisetosa

Taxon classificationAnimaliaDipteraMycetophilidae

Jaschhof & Hippa, 2005

##### Studied material.

COSTA RICA. 1 male, San Isidro de las Peñas Blancas, Texas A&M Soltis Center, Malaise trap, 420 m, 10°23'00"N, 84°36'58"W, 15.vi–10.vii.2010, Wendy Porras col. (on slide, MNCR).

##### Remarks.

*Manota multisetosa* was earlier known only from Costa Rica (Jaschhof and Hippa 2005) and Ecuador (Hippa and Kurina 2013).

#### Manota parva

Taxon classificationAnimaliaDipteraMycetophilidae

Jaschhof & Hippa, 2005

##### Studied material.

COLOMBIA. 2 males, Chocó, PNN Utría Boroboro, 06°01'S 77°20'W, 10 m, Malaise trap, 01–05.vii.2000, B. Brown Leg. M 3310 (on slide, 1 male IAvH, 1 male MZUSP); 1 male, Risaralda, SFF Otún Quimbaya El Molinillo, 04°43'N, 75°34'W, 2200 m, Malaise trap, 03–14.i.2003, G. López Leg. M. 3701 (on slide, IZBE); 1 male, same data as previous except 17.ii–04.iii.2003, M. 3696 (on slide, IZBE); 1 male, Cauca, PNN Gorgona, El Saman, 02°58'N, 78°11'W, 5 m, Malaise trap, 11.xi.2001–18.i.2002, H. Torres col., M 2791 (on slide, IAvH); 1 male, Nariño, R.N. La Planada, Parcela Olga, 01°15'N, 78°15'W, 1,850 m, Malaise trap, 16.vii–02.ix.2001, G. Oliva col., M 665 (on slide, MZUSP). COSTA RICA. 3 males, San Isidro de las Peñas Blancas, Texas A&M Soltis Center, Malaise trap, 420 m, 10°23'00"N, 84°36'58"W, 15.vi–10.vii.2010, Wendy Porras col. (on slides, 2 males MNCR, 1 male MZUSP,); 3 males, same data as previous except 20.iv–26.v.2010 (on slides, MNCR); 2 males, same data as previous except 13–20.iv.2010 (on slides, MZUSP); 1 male, same data as previous except sweeping, 18.viii.2010 (on slide, MZUSP).

##### Remarks.

*Manota parva* was earlier known only from Costa Rica (Jaschhof and Hippa 2005) and Ecuador (Hippa and Kurina 2013). The additional records make it one of the widespread species at the north-west corner of South America and Central America.

#### Manota pisinna

Taxon classificationAnimaliaDipteraMycetophilidae

Hippa & Kurina, 2013

##### Studied material.

BRAZIL. 1 male, State of Roraima, Caracarai (Vila Caicubi, Trilhada do Bacaba), 00°58'36.5"S, 62°06'08.7"W, Malaise trap #2, 10.ix.2011, Biffi, G. & Prado, L.R. cols. (on slide, MZUSP)

##### Remarks.

Having been described from French Guyana (Hippa and Kurina 2013), the species has subsequently been recorded from Peru (Hippa et al. 2017). Without the Peruvian record, the species would represent a typical Guyana Shield distribution. However, its presence in Iquitos makes it probably another widespread species at least in north-west South America.

#### Manota spinosa

Taxon classificationAnimaliaDipteraMycetophilidae

Jaschhof & Hippa, 2005

##### Studied material.

COLOMBIA. 1 male, Vaupés, Estación Biológica Mosiro-Itajura (Caparú), Igapo, 01°04'S 69°31'W, 60 m, Malaise trap, 17–24.xi.2003, J. Pinzón Leg. M 4434 (in alcohol, IAvH); 1 male, same data as previous except 24.xi–01.xii.2002, M 4437 (on slide, MZUSP).

##### Remarks.

The species was earlier known from Costa Rica (Jaschhof and Hippa 2005) and Peru (Hippa et al. 2017). This distribution is the same of that of *M. parva*, *M. acuminata*, *M. diversiseta*, and probably *M. squamulata*.

#### Manota squamulata

Taxon classificationAnimaliaDipteraMycetophilidae

Jaschhof & Hippa, 2005

##### Studied material.

COSTA RICA. 1 male, San Isidro de las Peñas Blancas, Texas A&M Soltis Center, Sweeping, 420 m, 10°23'00"N, 84°36'58"W, 13–18. viii.2010, D. Ament col. (on slide, MNCR).

##### Remarks.

Having been described from Costa Rica (Jaschhof and Hippa 2005), the species has subsequently been recorded from Ecuador (Hippa and Kurina 2013).

## Discussion

**Figure 7.**
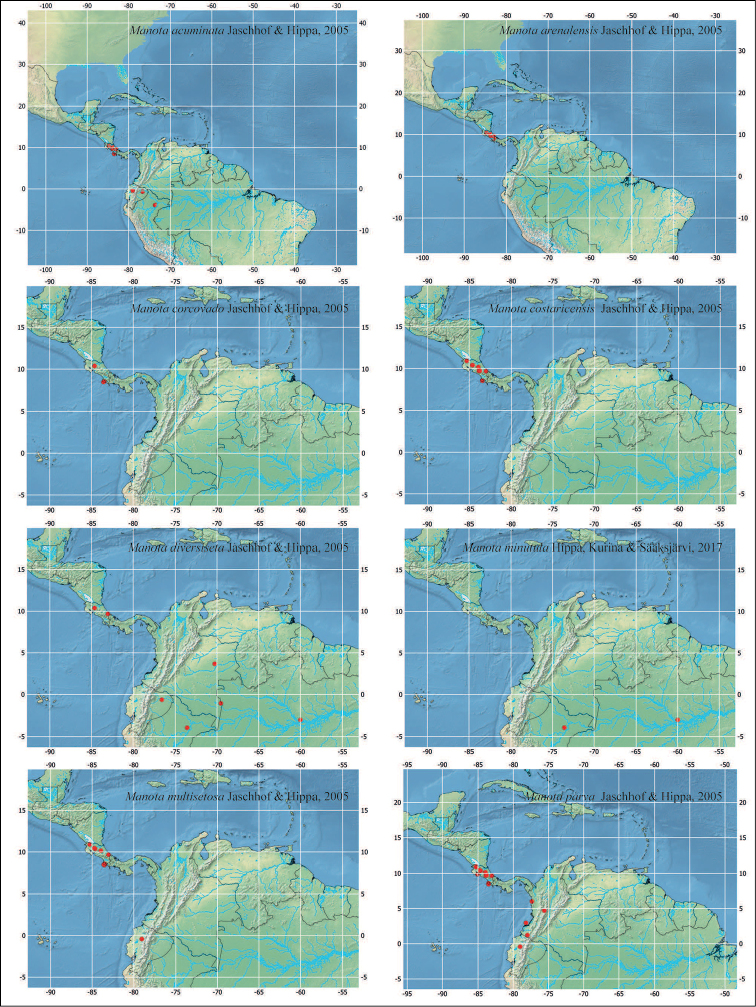
Distribution of described *Manota* species recorded in this paper.

**Figure 8.**
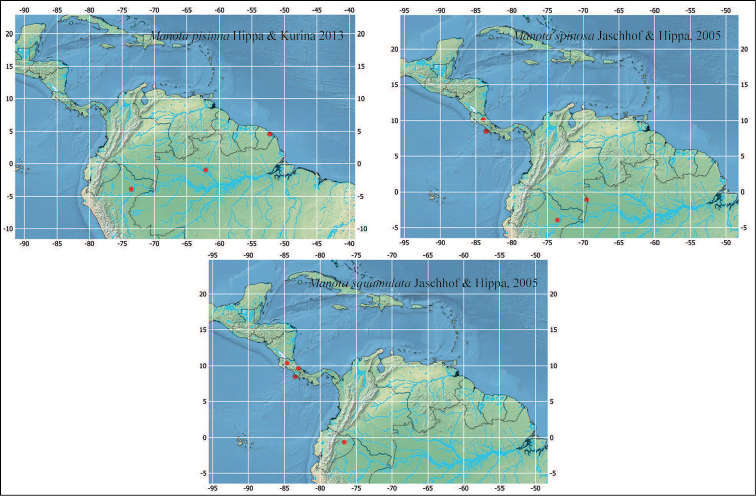
Distribution of described *Manota* species recorded in this paper.

A distribution map of the species described in this paper is depicted in Fig. 6, while maps in Figs 7–8 sum up the distribution of other recorded species including earlier data from Jaschhof and Hippa (2005), Hippa and Kurina (2012), and Hippa et al. (2017). Among the new species, only *M. subaristata*, sp. n. is known from more than one locality, all of them in Colombia. The other four new species are known only from the type locality. The species previously described accumulate more records that suggest some distribution patterns.

There are general patterns known for the Neotropical region (Amorim and Pires 1996, Amorim 2009) in which the fauna of north-western South America, including Amazon basin elements, connects to that of Central America. For some groups, as e.g. monkeys and some sciarids, as *Rhynchosciara* (Amorim and Pires 1996), the patterns refer to species restricted to smaller areas in lowlands on both sides of the Andes, Central America and Mexico, as well as in the Brazilian Amazon.

The observed *Manota* distribution patterns show individual species with a considerably wide distribution, which fit in this larger pattern—named as North-west Neotropical (Amorim 2009). In some cases, the species distribution is slightly more restricted and connects populations of lowlands in the Chocó region of Colombia, west to the Andes, to populations in Central America. This is a quite well-known pattern and in the genus *Manota* it is the case of *M. multisetosa* and *M. parva*. Future collections may show that this is either a real pattern or that these species actually have wider distributions and they just were still not found in other parts of South America. In other cases, species as *M. acuminata*, *M. diversiseta*, *M. spinosa*, and *M. squamulata*, present in Costa Rica, are also found east of the Andes, including Iquitos, at the west of the Amazon basin. This fits into an important biogeographical component, a triangular area delimited by the Andes, the Solimões river at the north, and Madeira-Mamoré rivers at the southeast. Some of the above-mentioned patterns can be discerned also in other groups like phorids of the genera *Apocephalus* Coquillett and *Dohrniphora* Dahl (Brown 2002, Brown and Kung 2007).

Although the patterns sometimes are obvious, explanations can be more complex. Nominal species distributions that enclose areas of different endemism may correspond to: (1) secondary expansion of younger species with prior local distribution; (2) lack of response of older species to barriers that affected the younger groups; or (3) clades with undetected, cryptic species. This cannot be answered for the *Manota* species in question. A phylogeographic study would be useful to verify whether populations of these widespread species at the extremes of their distribution are beyond the threshold of genetic differentiation, often used to recognized separate species. This kind of problem of insect species is hard to distinguish using only morphological features, as they can involve also “hidden” molecular divergence. This aspect has been addressed in a number of recent papers (e.g. Laamanen et al. 2003, Meier et al. 2006, Bickford et al. 2007, Tan et al. 2010, Rohner et al. 2014). If these taxonomical entities in *Manota* presently called species actually correspond to clades of more local species, the genus would show even more intensely its condition of an open-ended taxon (Bickel 2009). This would put *Manota* even closer to *Megaselia* as one of the most diverse and taxonomically complex genera in flies.

## Supplementary Material

XML Treatment for Manota

XML Treatment for Manota clava

XML Treatment for Manota multilobata

XML Treatment for Manota perplexa

XML Treatment for Manota setilobata

XML Treatment for Manota subaristata

XML Treatment for Manota acuminata

XML Treatment for Manota arenalensis

XML Treatment for Manota corcovado

XML Treatment for Manota costaricensis

XML Treatment for Manota diversiseta

XML Treatment for Manota minutula

XML Treatment for Manota multisetosa

XML Treatment for Manota parva

XML Treatment for Manota pisinna

XML Treatment for Manota spinosa

XML Treatment for Manota squamulata
